# Supplementary material for: Predictive radiomics for evaluation of cancer immune signature in glioblastoma: The PRECISE-GBM study
Source: Neurooncol Adv. 2026 May 2;8(1):vdag115. doi: 10.1093/noajnl/vdag115 (PMC13213608; doi:10.1093/noajnl/vdag115)
Supplement: vdag115_Supplementary_Data [file vdag115_supplementary_data.docx]

**PRECISE-GBM Supplementary Data File**

**Contents**

1. **Figures**

**Supplemental Figure S1**: (a) Workflow to match MRI and transcriptomic data from various databases for IDH-wildtype glioblastoma (b) MRI databases with corresponding MRI datasets along with synthetic FLAIR data and (c) transcriptomic data portals and corresponding datasets, utilized for curation of matched data followed by training and validation of PRECISE-GBM models **Supplemental Figure S2**: Preprocessing of MRI data and steps for extraction of radiomic features using DeepBraTumIA.

**Supplemental Figure S3**: Principal component analysis (PCA) of pre-harmonized radiomic data with color stratification

**Supplemental Figure S4:** Visualization of harmonized radiomics datapoints pre- and post-normalization using Z-score

**Supplemental Figure S5:** Cross-cohort held-out strategy.

**Supplemental Figure S6:** Examples of auto-segmented glioblastoma tumors

**Supplemental Figure S7**: Kaplan Meier survival curves for matched transcriptome and imaging datasets

**Supplemental Figure S8**: Pooled calibration curves for common and unique labels across pan-cancer and glioblastoma signatures

**Supplemental Figure S9**: Immune-related radiogenomic biomarkers for glioblastoma

1. **Tables:**

**Supplemental Table S1**: Immune category and the corresponding size of the selected radiomic feature set, showing feature contribution according to MRI sequence and tumor region

**Supplemental Table S2**: Open access links for imaging datasets

**Supplemental Table S3**: Cross-cohort hold-out validation results

**Supplemental Table S4**: Paired bootstrapped resampling for model comparison

**Supplemental Table S5**: GAN validation results

**Supplemental Table S6**: Checklist for Artificial Intelligence in Medical Imaging (CLAIM; 2024 Update)

1. **Python Libraries, Packages, Formulas**


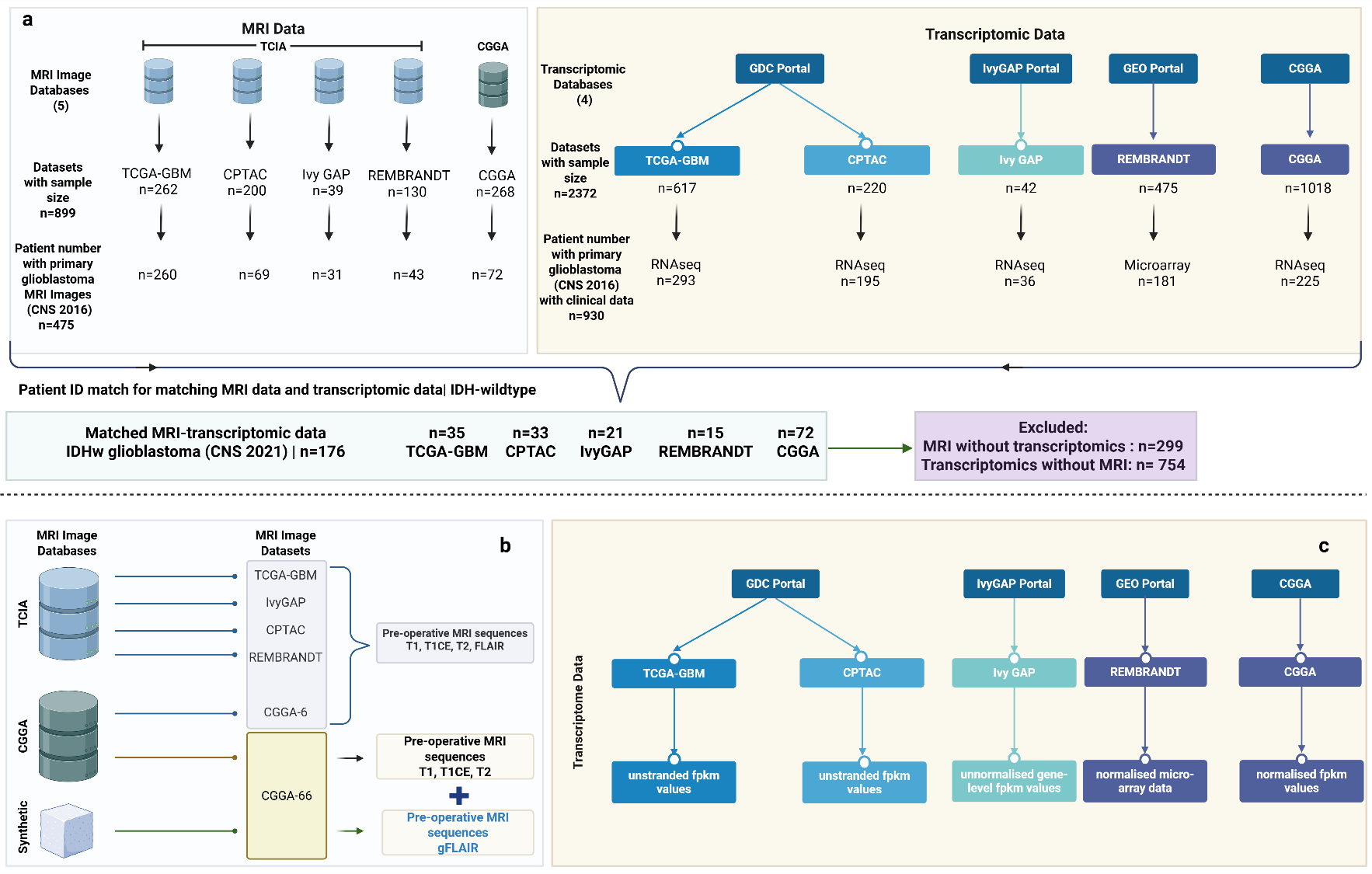


**Supplemental Figure S1**: (a) Workflow to match MRI and transcriptomic data from various databases for IDH-wildtype glioblastoma (b) MRI databases with corresponding MRI datasets along with synthetic FLAIR data and (c) transcriptomic data portals and corresponding datasets, utilized for curation of matched data followed by training and validation of PRECISE-GBM models. gFLAIR indicates generated FLAIR sequences using PRECISE-GAN model to supplement missing sequences.

*TCIA: The Cancer Imaging Archive, CGGA: Chinese Glioma Genome Atlas; TCGA-GBM: The Cancer Genome Atlas glioblastoma dataset; CPTAC: Clinical Proteomic Tumor Analysis Consortium; IvyGAP: Ivy Glioblastoma Atlas Project; REMBRANDT: REpository for Molecular BRAin Neoplasia DaTa; CGGA-6: 6 patients with MRI from CGGA dataset with all 4 sequences; CGGA-66: 66 patients with MRI images from CGGA dataset with missing FLAIR sequences requiring generation of synthetic FLAIR images; gFLAIR: generated FLAIR; GDC: Genomic Data Commons; GEO: Gene Expression Omnibus, IvyGAP: Ivy Glioblastoma Atlas Project; fpkm: fragments per kilobase of transcript per million fragments; T1: T1-weighted MRI sequence, T2: T2-weighted MRI sequence; FLAIR: fluid attenuated inversion recovery T2 MRI sequence; T1 CE: T1 MRI sequence with contrast enhancement; IDHw: IDH-wildtype.*


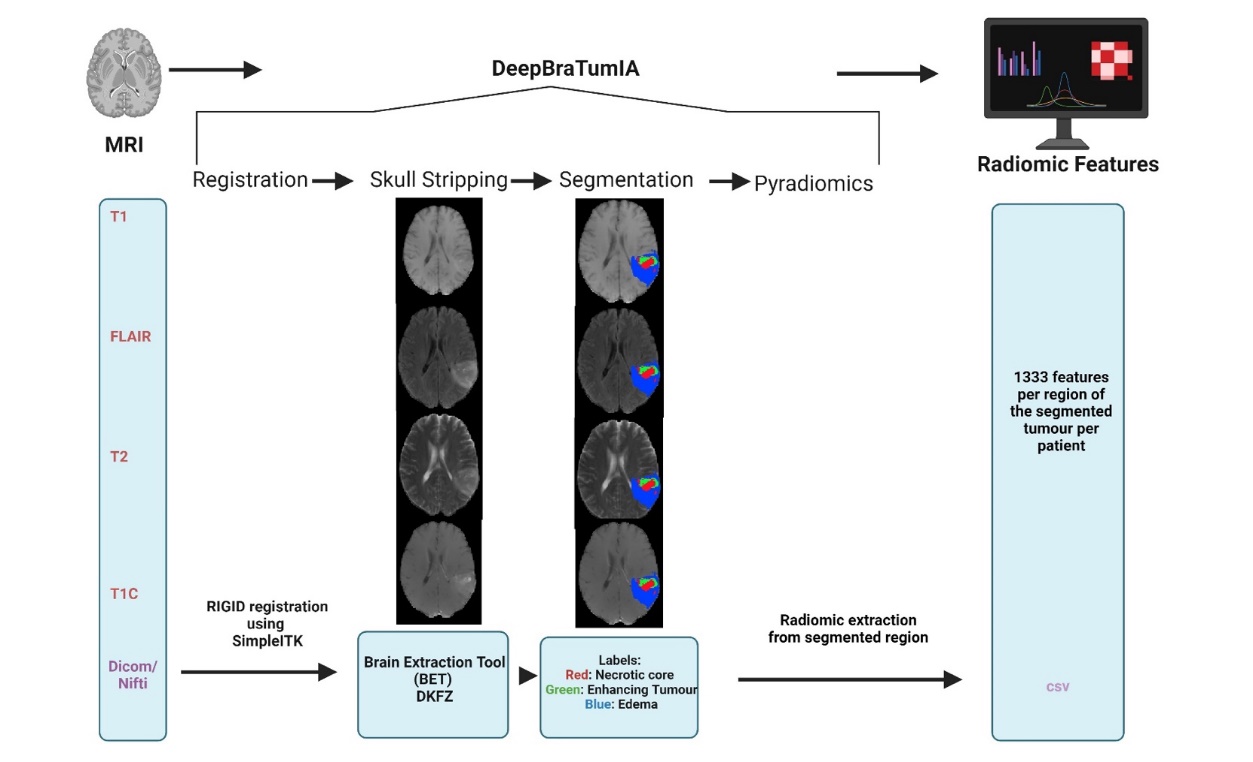


**Supplemental Figure S2**: Preprocessing of MRI data and steps for extraction of radiomic features using DeepBraTumIA (deep learning-based brain tumor image analysis).

*DKFZ: Deutsches Krebsforschungszentrum (German Cancer Research Centre, Heidelberg, Germany); SimpleITK: https://simpleitk.org/, RIGID registration to default MNI152_T1_1mm atlas; Skull stripping: https://github.com/MIC-DKFZ/HD-BET; Segmentation: https://github.com/CCI-Bonn/HD-GLIO-AUTO; T1: T1-weighted MRI sequence, T2: T2-weighted MRI sequence; FLAIR: fluid attenuated inversion recovery T2 MRI sequence; T1 CE: T1 MRI sequence with contrast enhancement.*

**
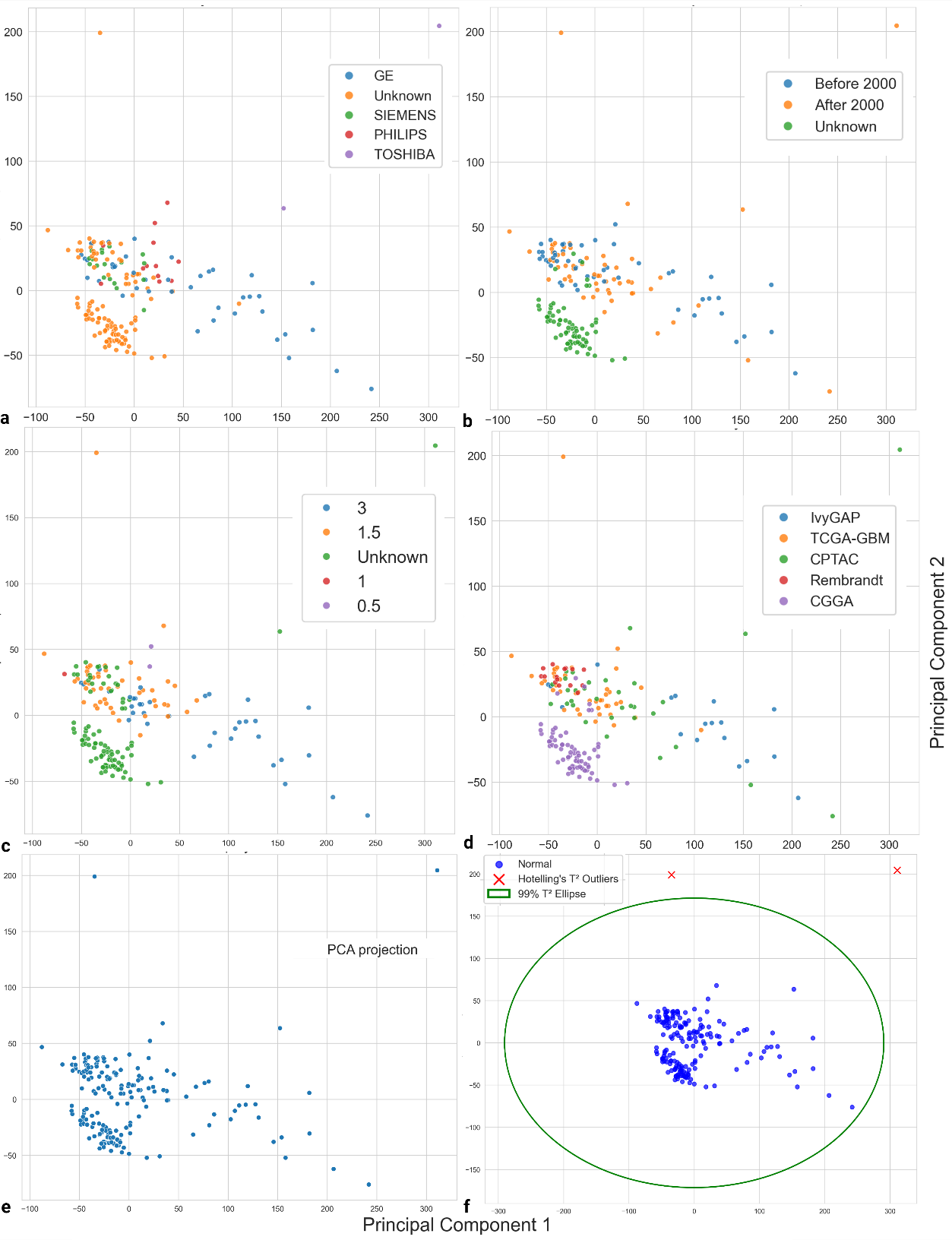
**

**Supplemental Figure S3:** Principal component analysis (PCA) of pre-harmonized radiomic data with color stratification according to (a) MRI manufacturer; (b) MRI year of acquisition; (c) MRI field strength; (d) datasets; (e) radiomics data without color stratification; (f) datapoints as outliers; PCA scatter plot of PC1 and PC2 with color code by using Hotelling’s T2 ellipse boundary of 99.9% confidence interval. CGGA dataset did not have any metadata available. We assigned the metadata outliers as the two patients who underwent TOSHIBA manufacturer scans of unknown Tesla strength from 2012 in the CPTAC dataset - C3L-01146, C3L-01156; and the one patient who underwent an unknown manufacturer scan of 1.5 T strength scanner from 2000 in the TCGA-GBM dataset - TCGA-06-0168.

*PC1: principal component 1; PC2: principal component 2; CGGA: Chinese Glioma Genome Atlas; TCGA-GBM: The Cancer Genome Atlas glioblastoma dataset; CPTAC: Clinical Proteomic Tumor Analysis Consortium; IvyGAP: Ivy Glioblastoma Atlas Project; REMBRANDT: REpository for Molecular BRAin Neoplasia DaTa*

**
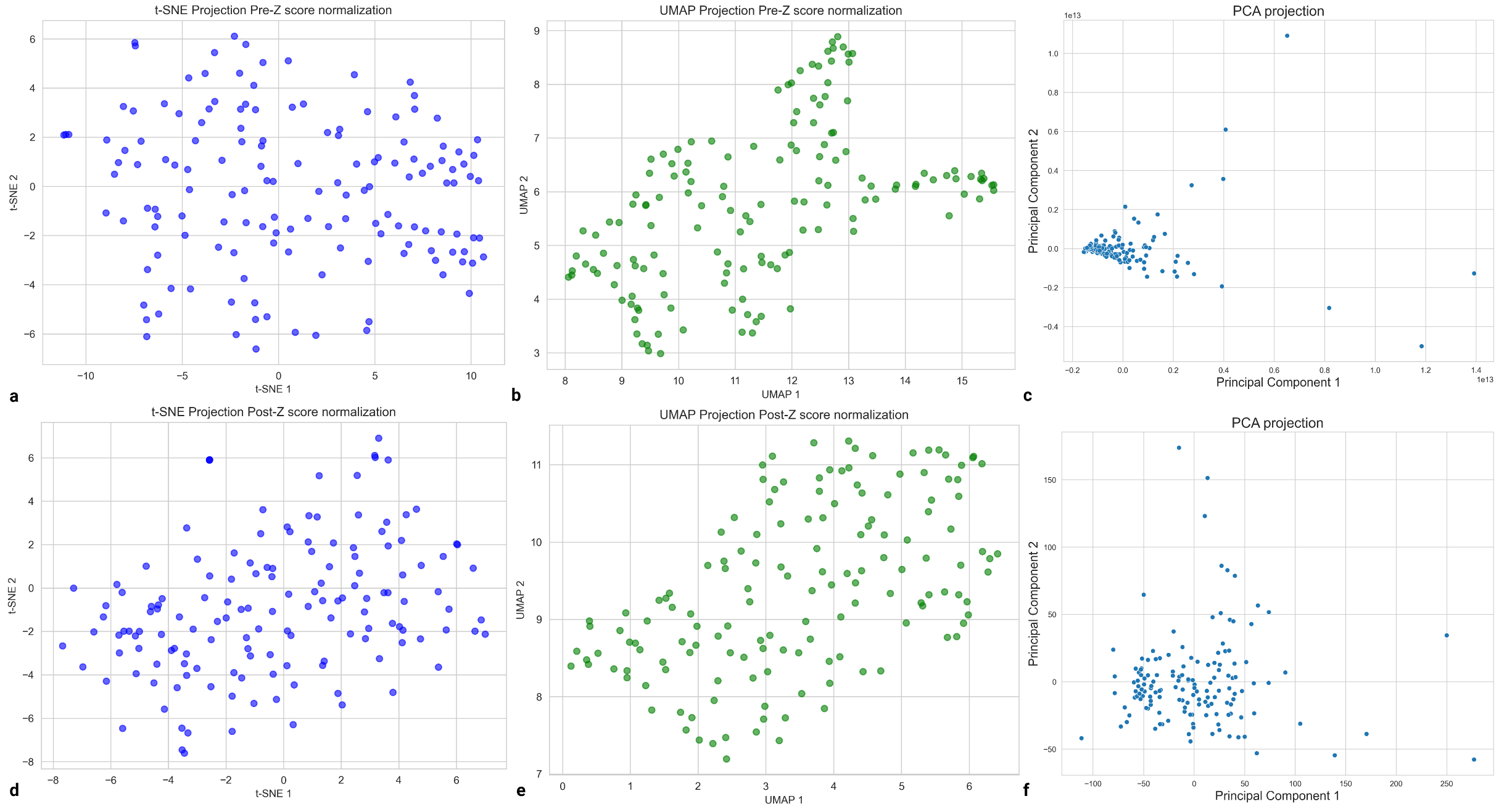
**

**Supplemental Figure S4:** Visualization of harmonized radiomics datapoints pre- (a, b, c) and post-normalization (d, e, f) using Z-score^1,2^. Example of Group 1 training data with visualization of the data points using a, d: t-SNE; b, e: UMAP and c, f: PCA scatter plots. *Please note that the clusters have changed since removal of PCA outliers and post-harmonization*.

*t-SNE: t-distributed stochastic neighbor embedding; UMAP: uniform manifold approximation and projection; PCA: principal component analysis*

**
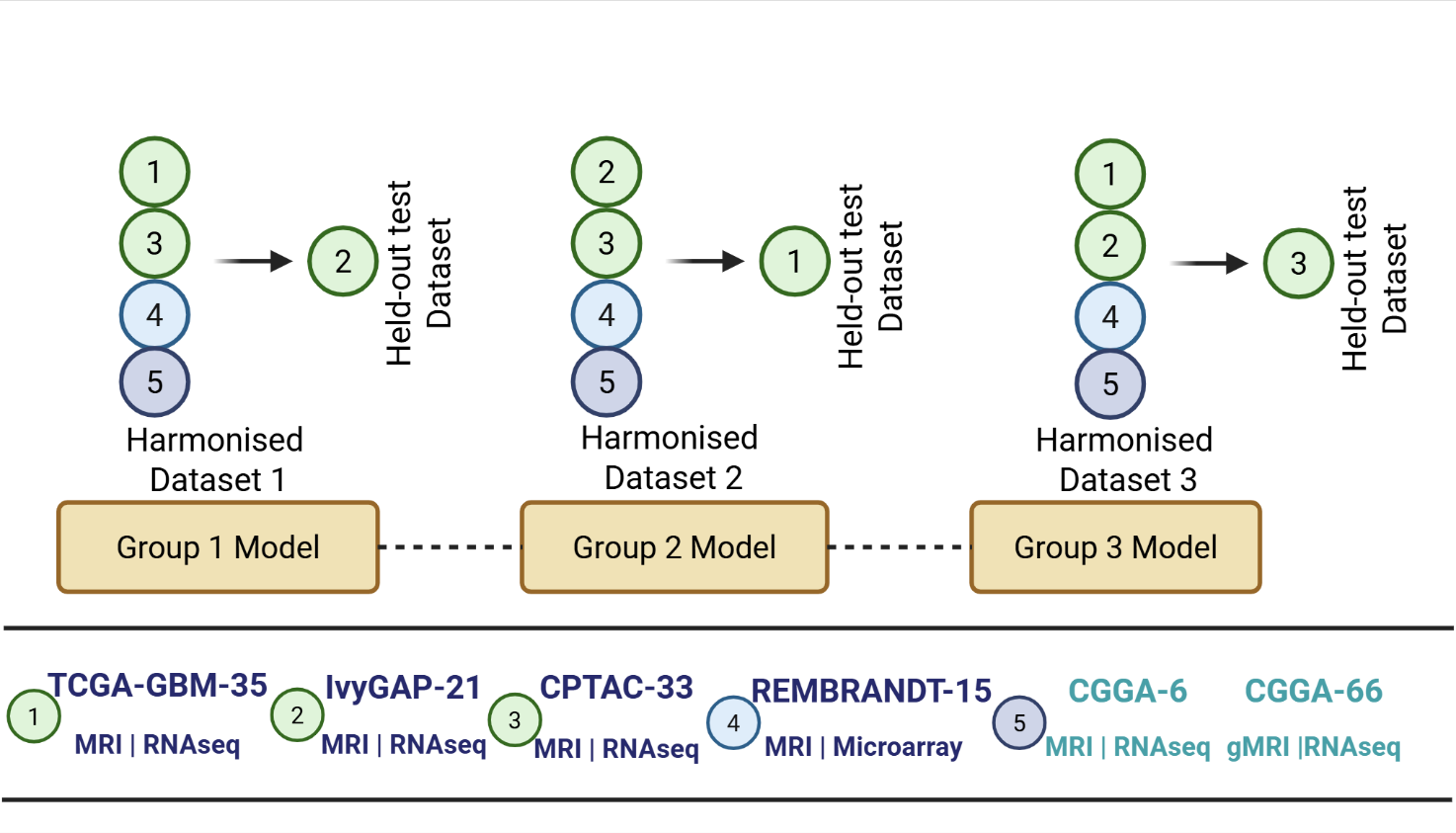
**

**Supplemental Figure S5**: Cross-cohort held-out strategy. The harmonized training dataset in each of the three Groups underwent Z score normalization followed by Least Absolute Shrinkage and Selection Operator (LASSO) with nested 5-fold CV for leakage free feature selection. Using the same training datasets, these selected features underwent classifier training in the form of 5-fold stratified cross validation with selection of hyperparameters and selection of the best trained model. Re-training using the best model and 100% of the training data (as opposed to 80%) gave the final fixed model which was tested on the respective held-out dataset. There was no data leakage.


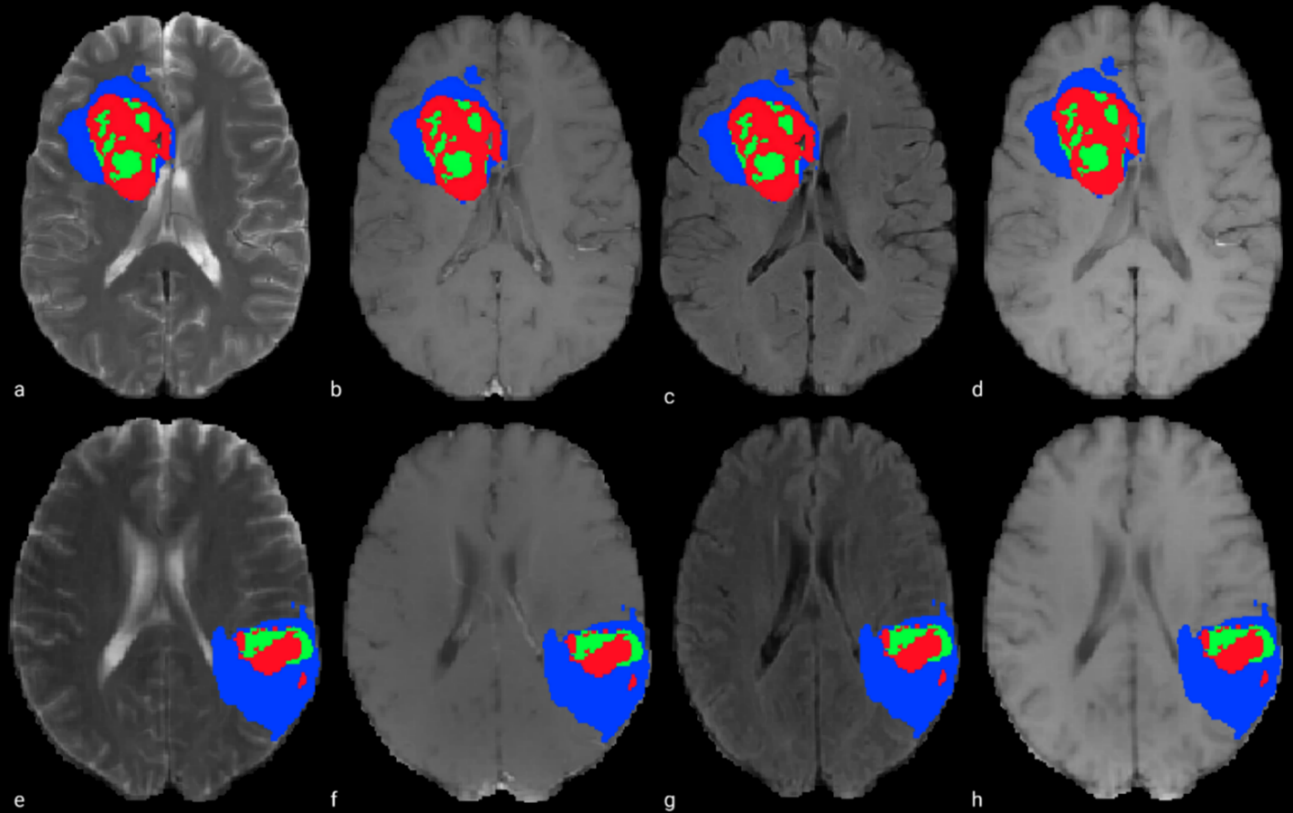


**Supplemental Figure S6:** Examples of auto-segmented glioblastoma tumors for patient 1 (a, b, c, d) with right frontal tumor, and patient 2 (e, f, g, h) with left parietal tumor. MRI sequences are *T*_2_-weighted (a, e), *T*_1_-weighted contrast-enhanced (b, f), *T*_2_ Fluid Attenuated Inversion Recovery (FLAIR) (c, g) and *T*_1_-weighted (d, h). The color codes are regions of the segmented tumor: necrotic core (NC): red; enhancing tumor (ET): green; “edema” (E): blue. Note that “edema” is in inverted commas to acknowledge that the nomenclature follows a segmentation naming convention, but the T2-weighted hyperintensity does not represent edema alone. Note that “necrotic core” is a combination of necrotic core and non-enhancing tumor in v2.1.0 DeepBraTumIA^35,36^ (University of Bern, Switzerland).


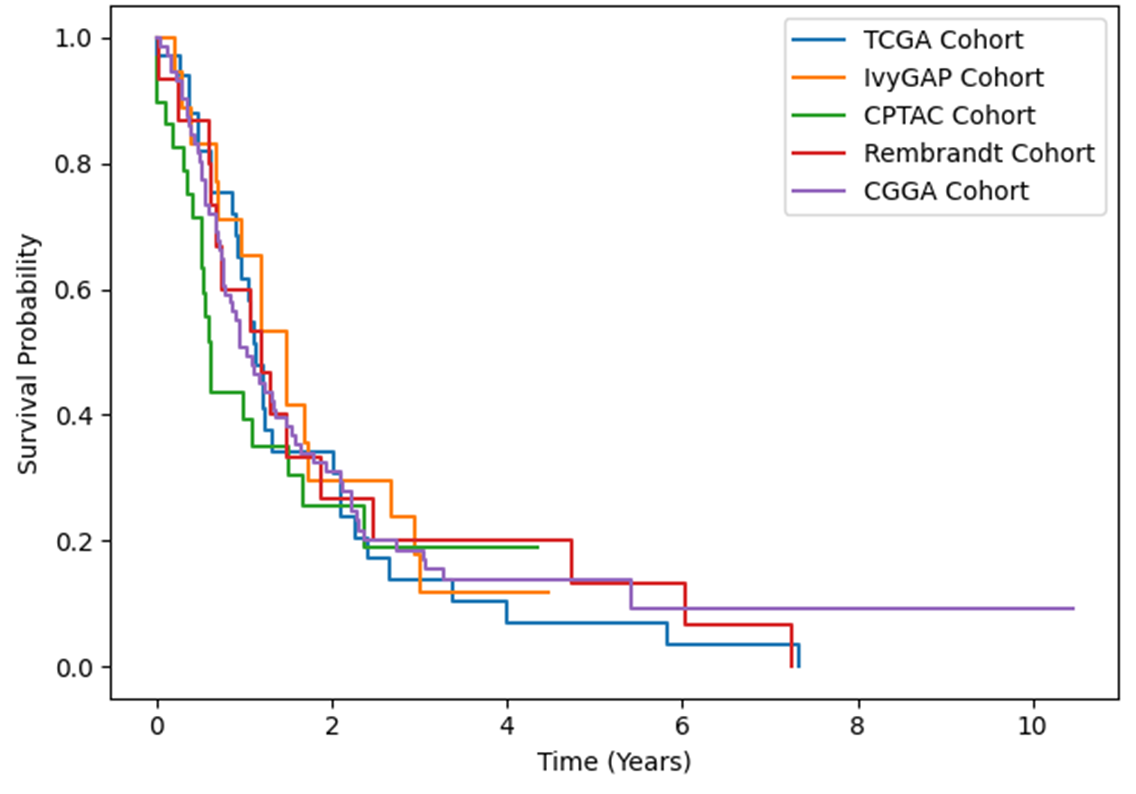
**Supplemental Figure S7**: Kaplan Meier survival curves for matched transcriptome and imaging datasets. There was no significant difference between the survival curves on log-rank tests (p-value < 0.05 considered statistically significant).


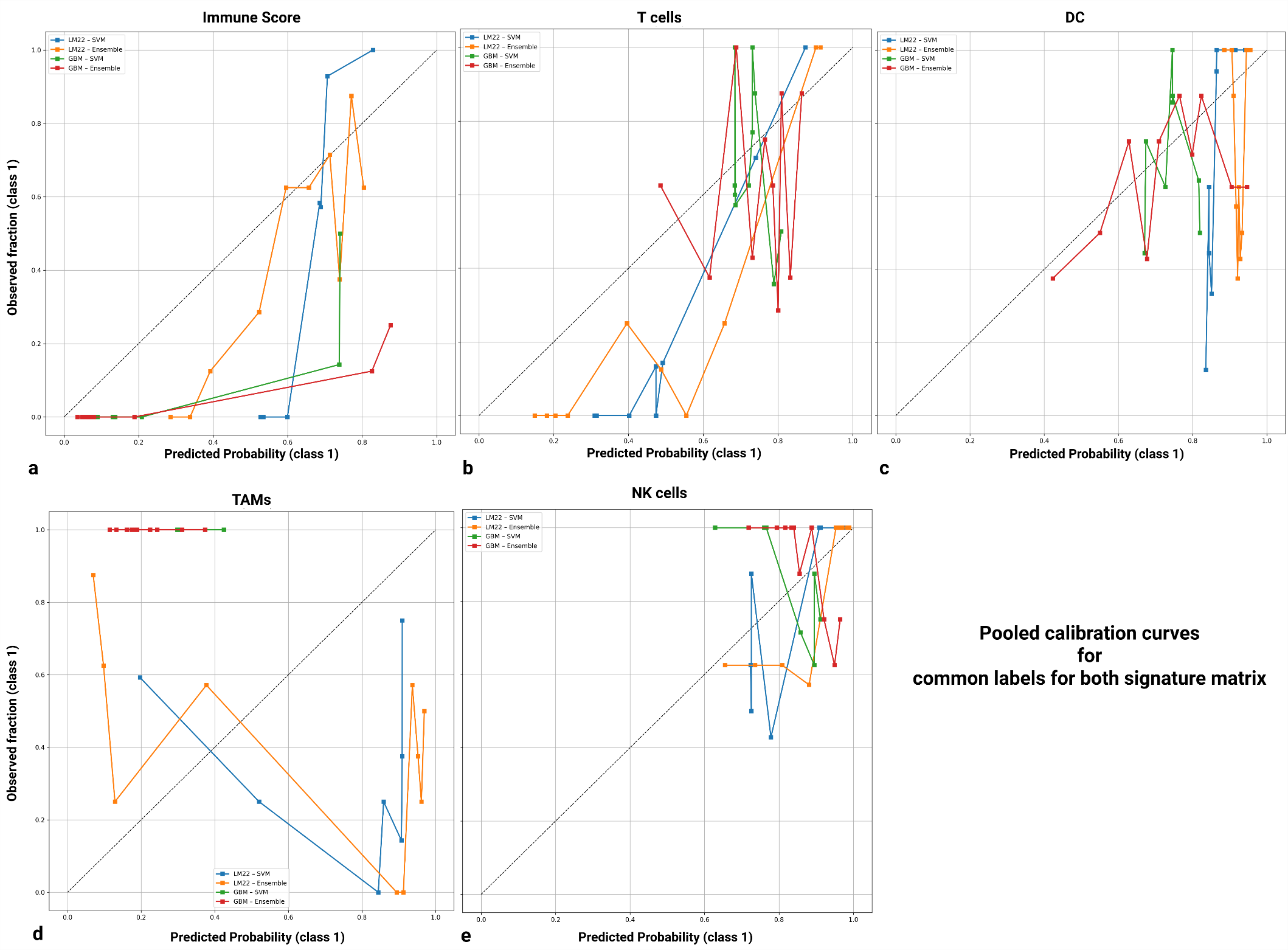


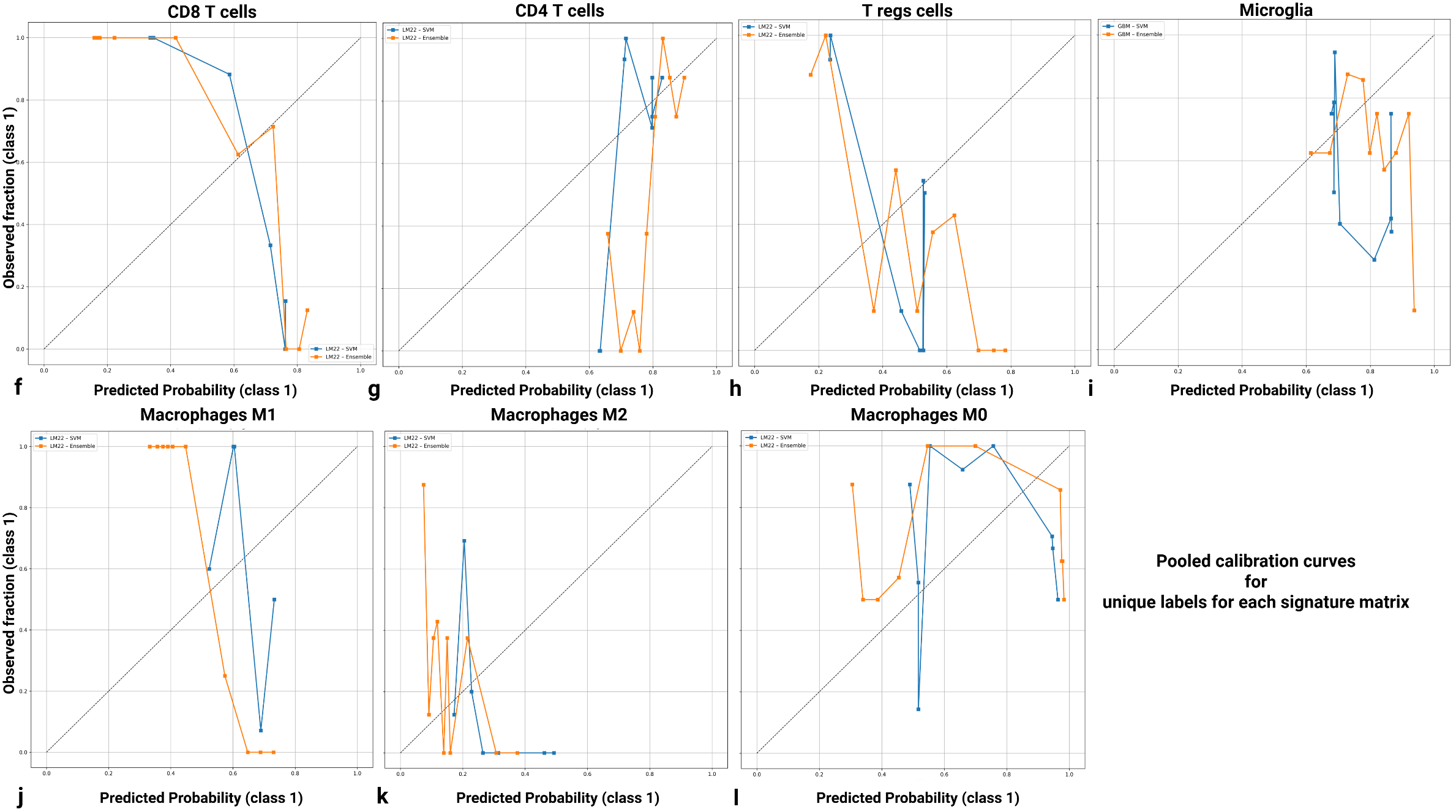


**Supplemental Figure S8:** Pooled calibration curves for common and unique labels across pan-cancer and glioblastoma signatures**.** Calibration curves were generated using held-out test predictions pooled across all evaluation groups 1-3. The x-axis shows the mean predicted probability of immune-high status (class 1) within probability bins, while the y-axis shows the observed fraction of immune-high cases in each bin. The dashed diagonal line represents perfect calibration.

Solid lines compare SVM and ensemble classifiers trained using pan-cancer(LM22) and glioblastoma (GBM) signatures for shared immune labels (**a**: Immune score, **b**: T cells, **c**: dendritic cells (DC), **d**: TAMs, **e**: natural killer cells (NK cells)) and unique immune labels (**f**: CD8 T cells, **g**: CD4 T cells, **h**: T regulatory cells (Tregs), **i**: microglia (GBM-specific), Macrophages **j**: M1, k: M2, **l**: M0).

Calibration quality is label dependent. For common labels, ensemble models generally exhibited smoother calibration profiles and reduced variance compared with SVMs, particularly for immune labels with sufficient prevalence. Deviations from the diagonal for certain labels reflect class imbalance and limited support in the held-out data rather than model instability.

For some unique labels, predicted probabilities often clustered at moderate to high values despite low observed event rates, resulting in apparent over-confidence due to substantial class imbalance. These patterns highlight the challenges of probabilistic calibration for rare immune populations and suggest that, for such labels, model outputs are better interpreted in a relative or ranking-based manner rather than an absolute probability estimates.These pooled analyses emphasize systematic calibration behaviour independent of group-specific splits and facilitate direct comparison between signatures and classifier families.

*SVM: support vector machine; TAMs: tumour associated macrophages*

*
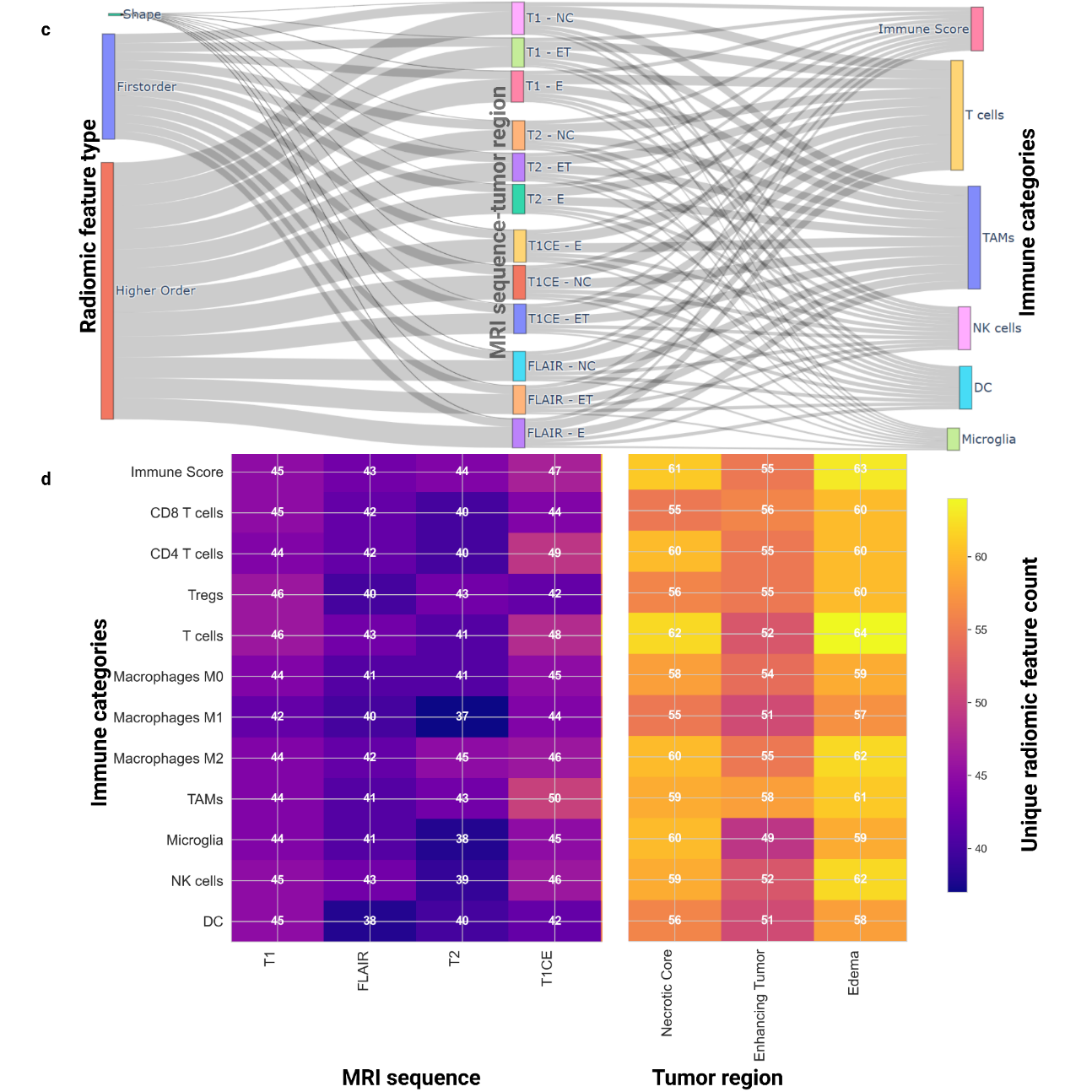

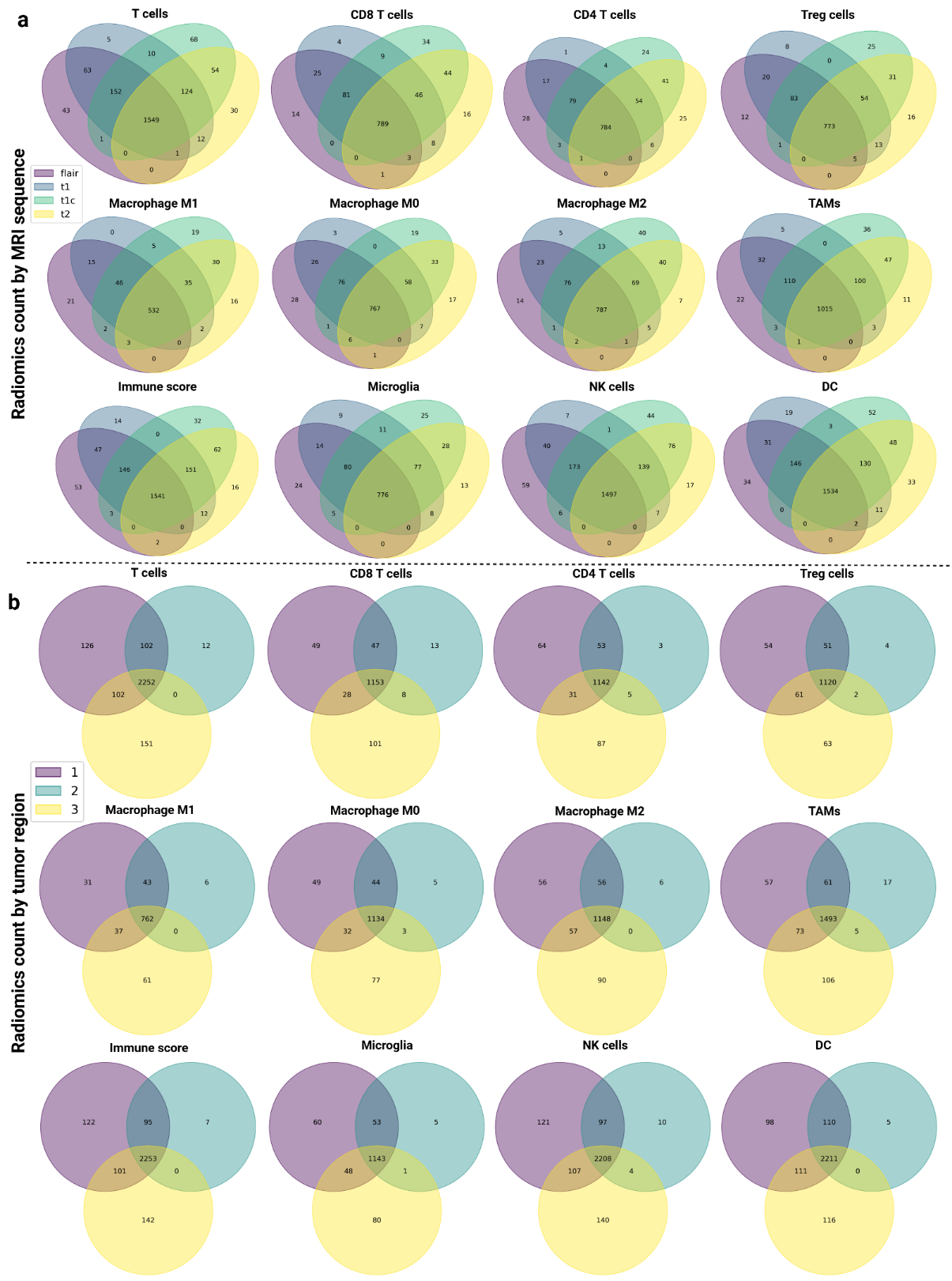
*

**Supplemental Figure S9: Immune-related radiogenomic biomarkers for glioblastoma**

a and b: Distribution of selected radiomic features for immune signatures in glioblastoma across (a) MRI sequences and (b) tumor regions,

Composite Venn diagrams illustrating the overlap and uniqueness of selected radiomic features associated with each immune category predicted by PRECISE-GBM models. For each immune signature, radiomic feature distributions are shown stratified by MRI sequences (a) (FLAIR, T1, T1 CE and T2) and tumor regions (b) (1 = necrotic core, 2 = enhancing tumor and 3 = edema region). The diagrams demonstrate that although several radiomic features are shared across multiple MRI sequences and tumor regions, distinct subsets of features remain sequence- and region-specific, indicating that different MRI contrasts and tumor compartments contribute complementary information for immune signature prediction.

c. Imaging-immune linkages in IDH-wildtype glioblastoma radiogenomic modeling. The Sankey diagram demonstrates the linkages of selected radiomic features across feature types, MRI sequences, tumor regions and immune categories complied together from 3 scenarios of feature selection of Group 1-3. Shape, first-order and higher order features are mapped onto FLAIR, T1, T2 and T1 CE sequences, localized to necrotic core (NC), enhancing tumor (ET) and “edema” regions (E), and contribute to immune categories (overall immune score, T cells, tumor associated macrophages (TAMs), dendritic cells (DC), natural killer (NK) cells and microglia cells). The contributions from MRI sequences and tumor regions are similar. The size of the linkage as well as the size of the bars represent the size of contribution from the respective components. Note that “edema” is in inverted commas to acknowledge that the nomenclature follows a segmentation naming convention, but the T2-weighted hyperintensity does not represent edema alone.

d. Immune-related radiomic features, whose frequency is color-coded in the heatmap, across various MRI sequences and tumor regions. The x-axis represents different MRI sequences (T1, FLAIR, T2, T1CE) and tumor regions - these two groups should be considered in isolation as two independent heatmaps; the y-axis represents immune categories.

T1: T1-weighted MRI sequence, T2: T2-weighted MRI sequence; FLAIR: fluid attenuated inversion recovery T2 MRI sequence; T1 CE: T1 MRI sequence with contrast enhancement; IDH: isocitrate dehydrogenase.

**B. Tables**

**Supplemental Table S1**: Immune category and the corresponding size of the selected radiomic feature set, showing feature contribution according to MRI sequence and tumor region (the features were selected from a total of 1333 radiomic features per MRI sequence per tumor region).

| Immune category | Total unique radiomic features | T1 | FLAIR | T2 | T1CE | Necrotic core | Enhancing tumor | Edema |
| --- | --- | --- | --- | --- | --- | --- | --- | --- |
| CD4 T cells | 175 | 44 | 42 | 40 | 49 | 60 | 55 | 60 |
| DC | 165 | 45 | 38 | 40 | 42 | 56 | 51 | 58 |
| Immune Score | 179 | 45 | 43 | 44 | 47 | 61 | 55 | 63 |
| Macrophages M0 | 171 | 44 | 41 | 41 | 45 | 58 | 54 | 59 |
| Macrophages M1 | 163 | 42 | 40 | 37 | 44 | 55 | 51 | 57 |
| Macrophages M2 | 177 | 44 | 42 | 45 | 46 | 60 | 55 | 62 |
| Microglia | 168 | 44 | 41 | 38 | 45 | 60 | 49 | 59 |
| NK cells | 173 | 45 | 43 | 39 | 46 | 59 | 52 | 62 |
| T cells | 178 | 46 | 43 | 41 | 48 | 62 | 52 | 64 |
| T cells CD8 | 171 | 45 | 42 | 40 | 44 | 55 | 56 | 60 |
| T cells regulatory (Tregs) | 171 | 46 | 40 | 43 | 42 | 56 | 55 | 60 |
| TAMs | 178 | 44 | 41 | 43 | 50 | 59 | 58 | 61 |

*DC: dendritic cells, NK cells: natural killer cells; TAMs: tumor associated macrophages; T1: T1-weighted MRI sequence, T2: T2-weighted MRI sequence; FLAIR: fluid attenuated inversion recovery T2 MRI sequence; T1 CE: T1 MRI sequence with contrast enhancement*

**Supplemental Table S2**: Open access links for imaging and genomic datasets

| Datasets | Imaging data links | Genomic Data links |
| --- | --- | --- |
| TCGA-GBM | https://www.cancerimagingarchive.net/collection/tcga-gbm/ | https://portal.gdc.cancer.gov/ |
| CPTAC | https://www.cancerimagingarchive.net/collection/cptac-gbm/ | https://portal.gdc.cancer.gov/ |
| IvyGAP | https://www.cancerimagingarchive.net/collection/ivygap/ | https://glioblastoma.alleninstitute.org/ |
| REMBRANDT | https://www.cancerimagingarchive.net/collection/rembrandt/ | https://www.ncbi.nlm.nih.gov/geo/query/acc.cgi?acc=GSE108474 |
| CGGA | https://www.cgga.org.cn/download.jsp | https://www.cgga.org.cn/download.jsp |

**Supplemental Table S3**: Cross-cohort hold-out validation results. Three sets of experiments were performed using three different combinations of training and holdout test datasets. These are referred to as Group 1, 2 and 3. Group 1, 2, and 3 holdout test results are presented in sub-tables a, b and c, respectively. Two transcriptome signature matrices (pan cancer and glioblastoma signature matrices) were used to obtain immune categories to be used as binary classification labels.

The metric results for the models with an asterisk were undefined due to collapse of the metric calculation due to extreme class imbalance in the held-out datasets (immune categories with only a single gaussian class or only a single patient in one of the gaussian classes of high versus low scores). Figure 4 in the main paper depicts these results in forest plots for all the groups.

*TAMs: tumor associated macrophages; NK cells: natural killer cells; SVM: support vector machine; DC: dendritic cells; MCC: mathews correlation coefficient where 1 indicates perfect classification, 0 represents random classification, -1 complete disagreement; ensemble models: ensemble of SVM, random forest (RF) and histogram-based gradient boost (GB) models; “-” : undefined.*

1. Group 1 hold-out test results (holdout dataset: IvyGAP)

| Pan cancer signature matrix | Immune Category | Radiogenomic model | Precision | Accuracy | Balanced accuracy | Recall | F1 score | MCC | Radiogenomic model | Precision | Accuracy | Balanced accuracy | Recall | F1 score | MCC |
| --- | --- | --- | --- | --- | --- | --- | --- | --- | --- | --- | --- | --- | --- | --- | --- |
|  | Immune score | SVM_immune_ | 0.94 | 0.94 | 0.50 | 1.00 | 0.97 | 0.0 | ENS_immune_ | 0.94 | 0.88 | 0.47 | 0.94 | 0.94 | -0.27 |
|  | CD4 T cells | SVM_CD4_ | 1.00 | 1.00 | 1.00 | 1.00 | 1.00 | 0.0 | ENS_CD4_ | 1.00 | 1.00 | 1.00 | 1.00 | 1.00 | 0.0 |
|  | CD8 T cells | SVM_CD8_ | 0.88 | 0.88 | 0.50 | 1.00 | 1.00 | 0.0 | ENS_CD8_ | 0.85 | 0.65 | 0.36 | 0.73 | 0.79 | -0.27 |
|  | T reg cells | SVM_Treg_* | 1.00 | - | 0.50 | - | - | - | ENS_Treg_* | 1.00 | - | 0.50 | - | - | - |
|  | T cells | SVM_Tcells_ | 1.00 | 1.00 | 1.00 | 1.00 | 1.00 | 0.0 | ENS_Tcells_ | 1.00 | 1.00 | 1.00 | 1.00 | 1.00 | 0.0 |
|  | Macrophage M1 | SVM_M1_* | - | - | - | 1.00 | - | - | ENS_M1_* | - | - | - | 1.00 | - | - |
|  | Macrophage M2 | SVM_M2_* | - | 0.94 | 0.94 | 1.00 | - | - | ENS_M2_ | 1.00 | 1.00 | 1.00 | 1.00 | 1.00 | 0.0 |
|  | Macrophage M0 | SVM_M0_ | 0.94 | 0.94 | 0.50 | 1.00 | 0.97 | 0.0 | ENS_M0_ | 1.00 | 0.88 | 0.94 | 0.88 | 0.93 | 0.39 |
|  | TAMs | SVM_TAMs_* | - | - | - | 1.00 | - | - | ENS_TAMs_* | - | - | - | 1.00 | - | - |
|  | Dendritic cells | SVM_DC_ | 1.00 | 1.00 | 1.00 | 1.00 | 1.00 | 0.0 | ENS_DC_ | 1.00 | 1.00 | 1.00 | 1.00 | 1.00 | 0.0 |
|  | NK cells | SVM_NK_ | 1.00 | 1.00 | 1.00 | 1.00 | 1.00 | 0.0 | ENS_NK_ | 1.00 | 1.00 | 1.00 | 1.00 | 1.00 | 0.0 |
| Glioblastoma signature matrix | Immune score | SVM_immune_ | 0.18 | 0.18 | 0.50 | 1.00 | 0.30 | 0.0 | ENS_immune_ | 0.18 | 0.18 | 0.50 | 1.00 | 0.30 | 0.0 |
|  | T cells | SVM_Tcells_ | 0.35 | 0.35 | 0.50 | 1.00 | 0.52 | 0.0 | ENS_Tcells_ | 0.35 | 0.35 | 0.50 | 1.00 | 0.52 | 0.0 |
|  | TAMs | SVM_TAMs_ | 0.12 | 0.12 | 0.50 | 1.00 | 0.21 | 0.0 | ENS_TAMs_ | 0.12 | 0.12 | 0.50 | 1.00 | 0.21 | 0.0 |
|  | Microglia | SVM_Micro_ | 0.94 | 0.94 | 0.50 | 1.00 | 0.97 | 0.0 | ENS_Micro_ | 0.94 | 0.94 | 0.50 | 1.00 | 0.97 | 0.0 |
|  | Dendritic cells | SVM_DC_ | 0.65 | 0.65 | 0.50 | 1.00 | 0.79 | 0.0 | ENS_DC_ | 0.65 | 0.65 | 0.50 | 1.00 | 0.79 | 0.0 |
|  | NK cells | SVM_NK_ | 1.00 | 1.00 | 1.00 | 1.00 | 1.00 | 0.0 | ENS_NK_ | 1.00 | 1.00 | 1.00 | 1.00 | 1.00 | 0.0 |

1. Group 2 hold-out test results (holdout dataset TCGA-GBM)

| Pan cancer signature matrix | Immune Category | Radiogenomic model | Precision | Accuracy | Balanced accuracy | Recall | F1 score | MCC | Radiogenomic model | Precision | Accuracy | Balanced accuracy | Recall | F1 score | MCC |
| --- | --- | --- | --- | --- | --- | --- | --- | --- | --- | --- | --- | --- | --- | --- | --- |
|  | Immune score | SVM_immune_ | 0.53 | 0.53 | 0.50 | 1.00 | 0.69 | 0.0 | ENS_immune_ | 0.53 | 0.53 | 0.50 | 1.00 | 0.69 | 0.0 |
|  | CD4 T cells | SVM_CD4_* | - | - | - | 1.00 | - | - | ENS_CD4_* | - | - | - | 1.00 | - | - |
|  | CD8 T cells | SVM_CD8_* | 1.00 | - | - | - | - | - | ENS_CD8_* | 1.00 | - | - | - | - | - |
|  | T reg cells | SVM_Treg_ | 0.38 | 0.44 | 0.57 | 1.00 | 0.55 | 0.23 | ENS_Treg_ | 0.41 | 0.56 | 0.58 | 1.00 | 0.50 | 0.20 |
|  | T cells | SVM_Tcells_ | 0.22 | 0.72 | 0.63 | 0.50 | 0.31 | 0.18 | ENS_Tcells_ | 0.06 | 0.41 | 0.34 | 0.25 | 0.10 | -0.19 |
|  | Macrophage M1 | SVM_M1_ | 1.00 | 1.00 | 1.00 | 1.00 | 1.00 | 0.0 | ENS_M1_ | 1.00 | 1.00 | 1.00 | 1.00 | 1.00 | 0.0 |
|  | Macrophage M2 | SVM_M2_ | 1.00 | 1.00 | 1.00 | 1.00 | 1.00 | 0.0 | ENS_M2_ | 1.00 | 1.00 | 1.00 | 1.00 | 1.00 | 0.0 |
|  | Macrophage M0 | SVM_M0_ | 0.67 | 0.67 | 0.50 | 1.00 | 0.79 | 0.0 | ENS_M0_ | 0.67 | 0.67 | 0.50 | 1.00 | 0.79 | 0.0 |
|  | TAMs | SVM_TAMs_ | 0.44 | 0.44 | 0.50 | 1.00 | 0.61 | 0.0 | ENS_TAMs_ | 0.44 | 0.44 | 0.50 | 1.00 | 0.61 | 0.0 |
|  | Dendritic cells | SVM_DC_ | 0.97 | 0.97 | 0.50 | 1.00 | 0.98 | 0.0 | ENS_DC_ | 0.97 | 0.97 | 0.50 | 1.00 | 0.98 | 0.0 |
|  | NK cells | SVM_NK_ | 1.00 | 1.00 | 1.00 | 1.00 | 1.00 | 0.0 | ENS_NK_ | 1.00 | 1.00 | 1.00 | 1.00 | 1.00 | 0.0 |
| Glioblastoma signature matrix | Immune score | SVM_immune_ | 1.00 | 1.00 | 1.00 | 1.00 | 1.00 | 0.0 | ENS_immune_ | 1.00 | 1.00 | 1.00 | 1.00 | 1.00 | 0.0 |
|  | T cells | SVM_Tcells_ | 0.61 | 0.59 | 0.48 | 0.95 | 0.74 | -0.14 | ENS_Tcells_ | 0.60 | 0.56 | 0.45 | 0.90 | 0.72 | -0.21 |
|  | TAMs | SVM_TAMs_ | 1.00 | 1.00 | 1.00 | 1.00 | 1.00 | 0.0 | ENS_TAMs_ | 1.00 | 1.00 | 1.00 | 1.00 | 1.00 | 0.0 |
|  | Microglia | SVM_Micro_ | 0.69 | 0.69 | 0.50 | 1.00 | 0.81 | 0.0 | ENS_Micro_ | 0.69 | 0.69 | 0.50 | 1.00 | 0.81 | 0.0 |
|  | Dendritic cells | SVM_DC_ | 0.47 | 0.47 | 0.50 | 1.00 | 0.64 | 0.0 | ENS_DC_ | 0.48 | 0.50 | 0.52 | 0.87 | 0.62 | 0.03 |
|  | NK cells | SVM_NK_ | 1.00 | 0.97 | 0.97 | 0.97 | 0.98 | 0.0 | ENS_NK_ | 1.00 | 1.00 | 1.00 | 1.00 | 1.00 | 0.0 |

1. Group 3 holdout test results (holdout dataset: CPTAC)

| Pan cancer signature matrix | Immune Category | Radiogenomic model | Precision | Accuracy | Balanced accuracy | Recall | F1 score | MCC | Radiogenomic model | Precision | Accuracy | Balanced accuracy | Recall | F1 score | MCC |
| --- | --- | --- | --- | --- | --- | --- | --- | --- | --- | --- | --- | --- | --- | --- | --- |
|  | Immune score | SVM_immune_* | - | - | - | 1.00 | - | - | ENS_immune_* | - | 0.83 | 0.83 | 1.00 | - | - |
|  | CD4 T cells | SVM_CD4_ | 0.79 | 0.79 | 0.50 | 1.00 | 0.89 | 0.0 | ENS_CD4_ | 0.79 | 0.79 | 0.50 | 1.00 | 0.89 | 0.0 |
|  | CD8 T cells | SVM_CD8_ | 0.10 | 0.10 | 0.50 | 1.00 | 0.19 | 0.0 | ENS_CD8_ | 0.10 | 0.10 | 0.50 | 1.00 | 0.19 | 0.0 |
|  | T reg cells | SVM_Treg_* | - | - | - | 1.00 | - | - | ENS_Treg_* | - | - | - | 1.00 | - | - |
|  | T cells | SVM_Tcells_ | 1.00 | 1.00 | 1.00 | 1.00 | 1.00 | 0.0 | ENS_Tcells_ | 1.00 | 1.00 | 1.00 | 1.00 | 1.00 | 0.0 |
|  | Macrophage M1 | SVM_M1_ | 1.00 | 1.00 | 1.00 | 1.00 | 1.00 | 0.0 | ENS_M1_ | 1.00 | 0.21 | 0.21 | 0.21 | 0.34 | 0.0 |
|  | Macrophage M2 | SVM_M2_* | 1.00 | 0.31 | 0.50 | - | - | - | ENS_M2_* | 1.00 | 0.31 | 0.50 | - | - | - |
|  | Macrophage M0 | SVM_M0_ | 0.61 | 0.59 | 0.47 | 0.94 | 0.74 | -0.15 | ENS_M0_ | 1.00 | 0.48 | 0.58 | 0.17 | 0.29 | 0.27 |
|  | TAMs | SVM_TAMs_* | 1.00 | 0.41 | 0.50 | - | - | - | ENS_TAMs_* | 1.00 | 0.41 | 0.50 | - | - | - |
|  | Dendritic cells | SVM_DC_ | 0.35 | 0.35 | 0.50 | 1.00 | 0.51 | 0.0 | ENS_DC_ | 0.35 | 0.35 | 0.50 | 1.00 | 0.51 | 0.0 |
|  | NK cells | SVM_NK_ | 0.59 | 0.59 | 0.50 | 1.00 | 0.74 | 0.0 | ENS_NK_ | 0.59 | 0.59 | 0.50 | 1.00 | 0.74 | 0.0 |
| Glioblastoma signature matrix | Immune score | SVM_immune_ | 1.00 | 1.00 | 1.00 | 1.00 | 1.00 | 0.0 | ENS_immune_ | 1.00 | 1.00 | 1.00 | 1.00 | 1.00 | 0.0 |
|  | T cells | SVM_Tcells_ | 0.79 | 0.76 | 0.48 | 0.96 | 0.86 | -0.10 | ENS_Tcells_ | 0.79 | 0.79 | 0.50 | 1.00 | 0.89 | 0.0 |
|  | TAMs | SVM_TAMs_* | 1.00 | - | - | - | - | - | ENS_TAMs_* | 1.00 | - | - | - | - | - |
|  | Microglia | SVM_Micro_ | 0.41 | 0.41 | 0.50 | 1.00 | 0.59 | 0.0 | ENS_Micro_ | 0.41 | 0.41 | 0.50 | 1.00 | 0.59 | 0.0 |
|  | Dendritic cells | SVM_DC_ | 0.86 | 0.86 | 0.50 | 1.00 | 0.93 | 0.0 | ENS_DC_ | 0.86 | 0.86 | 0.50 | 1.00 | 0.93 | 0.0 |
|  | NK cells | SVM_NK_ | 0.72 | 0.72 | 0.50 | 1.00 | 0.84 | 0.0 | ENS_NK_ | 0.72 | 0.72 | 0.50 | 1.00 | 0.84 | 0.0 |

**Supplemental Table S4**: Paired bootstrapped resampling for model comparison. Comparison was made of precision, balanced accuracy and MCC between model pairs (Model A vs Model B; 4 Models: SVM_pan , ENS_pan, SVM_gbm, ENS_gbm) generating 10,000 bootstrapped differences per comparison. In the 5 immune categories where there were all 4 models for the same immune category, 6 model pair combinations were determined, and a multiple comparison adjustment (Bonferroni) was applied. Raw p-values were calculated from the proportion of resamples where mean difference crossed 0. Model comparisons in immune score and T cell categories reached statistical significance for balanced accuracy. Immune categories without test results for any cohort in a group has been excluded as bootstrapping could not be performed; immune categories are only included if they have results from all three 3 groups for that metric.

*SVM: support vector machine; ENS: ensemble model; SVM_pan: support vector machine model trained with pan cancer signature matrix derived labels; SVM_gbm: support vector machine model trained with glioblastoma signature matrix derived labels; ENS_pan: ensemble model trained with pan cancer signature matrix derived labels; ENS_gbm: ensemble model trained with glioblastoma signature matrix derived labels; diff: difference; MCC: Mathews correlation coefficient; BA: Balanced accuracy; NK cells: natural killer cells; TAMs: tumor associated macrophages; NK cells: natural killer cells; T reg cells: T regulatory cells.*

**- statistically significant result post correction for multiple comparisons.

| Immune Category | Metric | Model A | Model B | Mean Difference | 95%  CI Lower | 95%  CI Upper | Raw  p-value | Bonferroni  p-value | Interpretation |
| --- | --- | --- | --- | --- | --- | --- | --- | --- | --- |
| DC | Precision | ENS_pan | ENS_gbm | 0.111524 | -0.51724 | 0.490489 | 0.5062 | 1 | ENS_pan favored; Not significant |
|  |  | ENS_pan | SVM_gbm | 0.11059 | -0.51724 | 0.5 | 0.5254 | 1 |  |
|  |  | SVM_gbm | ENS_gbm | -0.00321 | -0.00951 | 0 | 0.5902 | 1 | ENS_gbm favored; Not significant |
|  |  | SVM_pan | ENS_gbm | 0.112223 | -0.51724 | 0.490489 | 0.5078 | 1 | SVM_pan favored; Not significant |
|  |  | SVM_pan | ENS_pan | 0 | 0 | 0 | 1 | 1 | No difference favored; Not significant |
|  |  | SVM_pan | SVM_gbm | 0.112235 | -0.51724 | 0.5 | 0.5182 | 1 | SVM_pan favored; Not significant |
| Immune Score |  | SVM_gbm | ENS_gbm | 0 | 0 | 0 | 1 | 1 | No difference favored; Not significant |
| Macrophages M0 |  | SVM_pan | ENS_pan | -0.14982 | -0.39286 | 0 | 0.075 | 0.075 | ENS_pan favored; Not significant |
| Macrophages M2 |  | SVM_pan | ENS_pan | 0 | 0 | 0 | 1 | 1 | No difference favored; Not significant |
| Microglia |  | SVM_gbm | ENS_gbm | 0 | 0 | 0 | 1 | 1 |  |
| NK cells |  | ENS_pan | ENS_gbm | -0.04608 | -0.13793 | 0 | 0.592 | 1 | ENS_gbm favored; Not significant |
|  |  | ENS_pan | SVM_gbm | -0.04582 | -0.13793 | 0 | 0.5956 | 1 | SVM_gbm favored; Not significant |
|  |  | SVM_gbm | ENS_gbm | 0 | 0 | 0 | 1 | 1 | No difference favored; Not significant |
|  |  | SVM_pan | ENS_gbm | -0.04637 | -0.13793 | 0 | 0.586 | 1 | ENS_gbm favored; Not significant |
|  |  | SVM_pan | ENS_pan | 0 | 0 | 0 | 1 | 1 | No difference favored; Not significant |
|  |  | SVM_pan | SVM_gbm | -0.0466 | -0.13793 | 0 | 0.5904 | 1 | SVM_gbm favored; Not significant |
| T cells |  | ENS_pan | ENS_gbm | 0.112864 | -0.51442 | 0.647059 | 0.745 | 1 | ENS_pan favored; Not significant |
|  |  | ENS_pan | SVM_gbm | 0.106661 | -0.5504 | 0.647059 | 0.7364 | 1 |  |
|  |  | SVM_gbm | ENS_gbm | 0.00959 | -0.00739 | 0.03598 | 0.5938 | 1 | SVM_gbm favored; Not significant |
|  |  | SVM_pan | ENS_gbm | 0.170123 | -0.3547 | 0.647059 | 0.506 | 1 | SVM_pan favored; Not significant |
|  |  | SVM_pan | ENS_pan | 0.053528 | 0 | 0.159722 | 0.5896 | 1 |  |
|  |  | SVM_pan | SVM_gbm | 0.151624 | -0.39068 | 0.647059 | 0.5342 | 1 |  |
| TAMs |  | SVM_gbm | ENS_gbm | 0 | 0 | 0 | 1 | 1 | No difference favored; Not significant |
| DC | Balanced Accuracy | ENS_pan | ENS_gbm | 0.161438 | -0.01373 | 0.5 | 0.5898 | 1 | ENS_pan favored; Not significant |
|  |  | ENS_pan | SVM_gbm | 0.166967 | 0 | 0.5 | 0.595 | 1 |  |
|  |  | SVM_gbm | ENS_gbm | -0.00453 | -0.01373 | 0 | 0.6122 | 1 | ENS_gbm favored; Not significant |
|  |  | SVM_pan | ENS_gbm | 0.165168 | -0.01373 | 0.5 | 0.5726 | 1 | SVM_pan favored; Not significant |
|  |  | SVM_pan | ENS_pan | 0 | 0 | 0 | 1 | 1 | No difference favored; Not significant |
|  |  | SVM_pan | SVM_gbm | 0.16555 | 0 | 0.5 | 0.5992 | 1 | SVM_pan favored; Not significant |
| Immune Score |  | ENS_pan | ENS_gbm | -0.22491 | -0.5 | -0.03125 | 0 | 0 | ENS_gbm favored; Significant** |
|  |  | ENS_pan | SVM_gbm | -0.22257 | -0.5 | -0.03125 | 0 | 0 | SVM_gbm favored; Significant** |
|  |  | SVM_gbm | ENS_gbm | 0 | 0 | 0 | 1 | 1 | No difference favored; Not significant |
| Macrophages M0 |  | SVM_pan | ENS_pan | -0.16014 | -0.375 | 0 | 0.0752 | 0.0752 | ENS_pan favored; Not significant |
| Macrophages M2 |  | SVM_pan | ENS_pan | 0 | 0 | 0 | 1 | 1 | No difference favored; Not significant |
| Microglia |  | SVM_gbm | ENS_gbm | 0 | 0 | 0 | 1 | 1 |  |
| NK cells |  | ENS_pan | ENS_gbm | 0 | 0 | 0 | 1 | 1 |  |
|  |  | ENS_pan | SVM_gbm | 0.010469 | 0 | 0.03125 | 0.5728 | 1 | ENS_pan favored; Not significant |
|  |  | SVM_gbm | ENS_gbm | -0.01055 | -0.03125 | 0 | 0.5746 | 1 | ENS_gbm favored; Not significant |
|  |  | SVM_pan | ENS_gbm | 0 | 0 | 0 | 1 | 1 | No difference favored; Not significant |
|  |  | SVM_pan | ENS_pan | 0 | 0 | 0 | 1 | 1 |  |
|  |  | SVM_pan | SVM_gbm | 0.010421 | 0 | 0.03125 | 0.5876 | 1 | SVM_pan favored; Not significant |
| T cells |  | ENS_pan | ENS_gbm | 0.312671 | -0.05952 | 0.5 | 0.0758 | 0.4548 | ENS_pan favored; Not significant |
|  |  | ENS_pan | SVM_gbm | 0.301017 | -0.11786 | 0.521739 | 0.0804 | 0.4824 | ENS_pan favored; Not significant |
|  |  | SVM_gbm | ENS_gbm | 0.012264 | -0.02174 | 0.058333 | 0.5954 | 1 | SVM_gbm favored; Not significant |
|  |  | SVM_pan | ENS_gbm | 0.404829 | 0.208333 | 0.5 | 0 | 0 | SVM_pan favored; Significant** |
|  |  | SVM_pan | ENS_pan | 0.090509 | 0 | 0.267857 | 0.596 | 1 | SVM_pan favored; Not significant |
|  |  | SVM_pan | SVM_gbm | 0.389875 | 0.15 | 0.521739 | 0 | 0 | SVM_pan favored; Significant** |
| CD8 T cells |  | SVM_pan | ENS_pan | 0.067507 | 0 | 0.2 | 0.5778 | 0.5778 | SVM_pan favored; Not significant |
| T reg cells |  | SVM_pan | ENS_pan | -0.01089 | -0.03247 | 0 | 0.5818 | 0.5818 | ENS_pan favored; Not significant |
| CD4 T cells | MCC | SVM_pan | ENS_pan | 0 | 0 | 0 | 1 | 1 | No difference favored; Not significant |
| DC |  | ENS_pan | ENS_gbm | -0.01025 | -0.03047 | 0 | 0.5814 | 1 | ENS_gbm favored; Not significant |
|  |  | ENS_pan | SVM_gbm | 0 | 0 | 0 | 1 | 1 | No difference favored; Not significant |
|  |  | SVM_gbm | ENS_gbm | -0.01013 | -0.03047 | 0 | 0.5948 | 1 | ENS_gbm favored; Not significant |
|  |  | SVM_pan | ENS_gbm | -0.01026 | -0.03047 | 0 | 0.5866 | 1 |  |
|  |  | SVM_pan | ENS_pan | 0 | 0 | 0 | 1 | 1 | No difference favored; Not significant |
|  |  | SVM_pan | SVM_gbm | 0 | 0 | 0 | 1 | 1 |  |
| Immune Score |  | ENS_pan | ENS_gbm | -0.02101 | -0.0625 | 0 | 0.59 | 1 | ENS_gbm favored; Not significant |
|  |  | ENS_pan | SVM_gbm | -0.02069 | -0.0625 | 0 | 0.5974 | 1 | SVM_gbm favored; Not significant |
|  |  | SVM_gbm | ENS_gbm | 0 | 0 | 0 | 1 | 1 | No difference favored; Not significant |
|  |  | SVM_pan | ENS_gbm | 0 | 0 | 0 | 1 | 1 |  |
|  |  | SVM_pan | ENS_pan | 0.020783 | 0 | 0.0625 | 0.5882 | 1 | SVM_pan favored; Not significant |
|  |  | SVM_pan | SVM_gbm | 0 | 0 | 0 | 1 | 1 | No difference favored; Not significant |
| Macrophages M0 |  | SVM_pan | ENS_pan | -0.26764 | -0.41328 | 0 | 0.0732 | 0.0732 | ENS_pan favored; Not significant |
| Macrophages M1 |  | SVM_pan | ENS_pan | 0 | 0 | 0 | 1 | 1 | No difference favored; Not significant |
| Macrophages M2 |  | SVM_pan | ENS_pan | 0 | 0 | 0 | 1 | 1 |  |
| Microglia |  | SVM_gbm | ENS_gbm | 0 | 0 | 0 | 1 | 1 |  |
| NK cells |  | ENS_pan | ENS_gbm | 0 | 0 | 0 | 1 | 1 |  |
|  |  | ENS_pan | SVM_gbm | 0 | 0 | 0 | 1 | 1 |  |
|  |  | SVM_gbm | ENS_gbm | 0 | 0 | 0 | 1 | 1 |  |
|  |  | SVM_pan | ENS_gbm | 0 | 0 | 0 | 1 | 1 |  |
|  |  | SVM_pan | ENS_pan | 0 | 0 | 0 | 1 | 1 |  |
|  |  | SVM_pan | SVM_gbm | 0 | 0 | 0 | 1 | 1 |  |
| T cells |  | ENS_pan | ENS_gbm | 0.005846 | 0 | 0.017742 | 0.603 | 1 | ENS_pan favored; Not significant |
|  |  | ENS_pan | SVM_gbm | 0.015459 | -0.04986 | 0.096523 | 0.8288 | 1 |  |
|  |  | SVM_gbm | ENS_gbm | -0.00906 | -0.09652 | 0.067603 | 0.8228 | 1 | ENS_gbm favored; Not significant |
|  |  | SVM_pan | ENS_gbm | 0.131091 | 0 | 0.390617 | 0.5864 | 1 | SVM_pan favored; Not significant |
|  |  | SVM_pan | ENS_pan | 0.126181 | 0 | 0.372875 | 0.578 | 1 |  |
|  |  | SVM_pan | SVM_gbm | 0.140239 | 0 | 0.323014 | 0.0748 | 0.4488 |  |
| CD8 T cells |  | SVM_pan | ENS_pan | 0.090469 | 0 | 0.26968 | 0.5948 | 0.5948 |  |
| T reg cells |  | SVM_pan | ENS_pan | 0.011847 | 0 | 0.035396 | 0.5862 | 0.5862 |  |
| TAMs |  | ENS_pan | ENS_gbm | 0 | 0 | 0 | 1 | 1 | No difference favored; Not significant |
|  |  | ENS_pan | SVM_gbm | 0 | 0 | 0 | 1 | 1 |  |
|  |  | SVM_gbm | ENS_gbm | 0 | 0 | 0 | 1 | 1 |  |
|  |  | SVM_pan | ENS_gbm | 0 | 0 | 0 | 1 | 1 |  |
|  |  | SVM_pan | ENS_pan | 0 | 0 | 0 | 1 | 1 |  |
|  |  | SVM_pan | SVM_gbm | 0 | 0 | 0 | 1 | 1 |  |

**Supplemental Table S5:** GAN validation results. Training of PRECISE-GAN model included 76942 2D images from 3T pre-operative glioma T2 and FLAIR MRI of 490 patients and was validated on 785 2D images from 3T pre-operative glioma T2 and FLAIR MRI of 5 patients. 3D images were converted to 2D for each slice, images were resized to 256 x 256 pixels and intensity was normalized to [-1, 1] range as the pre-processing steps. The model was trained for 300 epochs with data augmentation and Adam optimizer with 2 losses namely GAN loss, L1 loss adding up to total generator loss. The model was trained using NVIDIA GeForce RTX 3080. MSE, SSI, PSNR and SSIM were the metrics for validation. The model achieved reconstruction quality useful for extraction of radiomics. *GAN: generative adversarial network, PRECISE-GAN:* *Production of reconstructed complete imaging using synthetic engineering for glioma through generative adversarial network , FLAIR: fluid attenuated inversion recovery; MSE: mean squared error; SSI: signal similarity index; PSNR: peak signal to noise ratio; SSIM: structural similarity index measure*

| PRECISE-GAN | Mean Validation Metrics (Range) | acceptable range of metric^3-6^ |
| --- | --- | --- |
| SSIM | 0.91 (0.906-0.914) | 0.89-0.91 |
| PSNR (dB) | 24.08 (23.37-24.47) | 20-30 |
| SSI | 0.84 (0.83-0.85) | 0.82-0.84 |
| MSE | 0.05 (0.01-0.14) | 0.03-0.07 |

**Supplemental Table S6**: Checklist for Artificial Intelligence in Medical Imaging (CLAIM; 2024)^7^

| Section / Topic | No. | Item | Page | No | | NA |
| --- | --- | --- | --- | --- | --- | --- |
| TITLE / ABSTRACT |  |  |  |  | |  |
|  | **1** | Identification as a study of AI methodology, specifying the category of technology used (e.g., deep learning) | **1, 2** |  | |  |
| ABSTRACT |  |  |  |  | |  |
|  | **2** | Summary of study design, methods, results, and conclusions | **2,3** |  | |  |
| INTRODUCTION |  |  |  |  | |  |
|  | **3** | Scientific and/or clinical background, including the intended use and role of the AI approach | **4,5** |  | |  |
|  | **4** | Study aims, objectives, and hypotheses | **4,5** |  | |  |
| METHODS |  |  |  |  | |  |
| *Study Design* | **5** | Prospective or retrospective study | **5** |  | |  |
|  | **6** | Study goal | **5** |  | |  |
| *Data* | **7** | Data sources | **5,6,7** |  | |  |
|  | **8** | Inclusion and exclusion criteria | **5,6,7** |  | |  |
|  | **9** | Data pre-processing | **6,7,8,9** |  | |  |
|  | **10** | Selection of data subsets | **5,6,7** |  | |  |
|  | **11** | De-identification methods | **5, 6,7** |  | |  |
|  | **12** | How missing data were handled | **6** |  | |  |
|  | **13** | Image acquisition protocol |  |  | | **N/A** |
| *Reference Standard* | **14** | Definition of method(s) used to obtain reference standard | **6, 7** |  | |  |
|  | **15** | Rationale for choosing the reference standard | **6,7** |  | |  |
|  | **16** | Source of reference standard annotations | **6,7,8** |  | |  |
|  | **17** | Annotation of test set | **6-11** |  | |  |
|  | **18** | Measures of inter- and intra-rater variability of features described by the annotators | **-** | **-** | | **N/A** |
| *Data Partitions* | **19** | How data were assigned to partitions | **6-10** |  | |  |
|  | **20** | Level at which partitions are disjoint | **-** | **-** | | **N/A** |
| *Testing Data* | **21** | Intended sample size | **11** |  | |  |
| *Model* | **22** | Detailed description of model | **9** |  |  | |
|  | **23** | Software libraries, frameworks, and packages | **6-11, Suppl. File** |  |  | |
|  | **24** | Initialization of model parameters | **9-11** |  |  | |
| *Training* | **25** | Details of training approach | **10,11** |  |  | |
|  | **26** | Method of selecting the final model | **11,12** |  |  | |
|  | **27** | Ensembling techniques | **9,10** |  |  | |
| *Evaluation* | **28** | Metrics of model performance | **9-11** |  |  | |
|  | **29** | Statistical measures of significance and uncertainty | **9-11** |  |  | |
|  | **30** | Robustness or sensitivity analysis | **9-11** |  |  | |
|  | **31** | Methods for explainability or interpretability | **10** |  |  | |
|  | **32** | Evaluation on internal data | **9, 10** |  |  | |
|  | **33** | Testing on external data | **9, 10** |  |  | |
|  | **34** | Clinical trial registration | **-** | **-** | **N/A** | |
| RESULTS |  |  |  |  |  | |
| *Data* | **35** | Numbers of patients or examinations included and excluded | **11** |  |  | |
|  | **36** | Demographic and clinical characteristics of cases in each partition | **11** |  |  | |
| *Model performance* | **37** | Performance metrics and measures of statistical uncertainty | **12-15** |  |  | |
|  | **38** | Estimates of diagnostic performance and their precision | **13-15** |  |  | |
|  | **39** | Failure analysis of incorrect results | **15** |  |  | |
| DISCUSSION |  |  |  |  |  | |
|  | **40** | Study limitations | **19-20** |  |  | |
|  | **41** | Implications for practice, including intended use and/or clinical role | **17-19** |  |  | |
| OTHER INFORMATION |  |  |  |  |  | |
|  | **42** | Provide a reference to the full study protocol or to additional technical details | **Suppl. file** |  |  | |
|  | **43** | Statement about the availability of software, trained model, and/or data | **24,25** |  |  | |
|  | **44** | Sources of funding and other support; role of funders | **24** |  |  | |

* Indicate page and/or line number for each checklist item that is present. NA = not applicable.

**Supplemental Table S7: TRIPOD-AI Checklist^8^**

| **Section/Topic Item Development Checklist item**  **/ evaluation**^1^ | | | | **Reported on page** |
| --- | --- | --- | --- | --- |
| **TITLE** | | | |  |
| *Title* | 1 | D;E | Identify the study as developing or evaluating the performance of a multivariable prediction model, the target population, and the outcome to be predicted | 1 |
| **ABSTRACT** | | | | |
| *Abstract* | 2 | D;E | TRIPOD+AI for Abstracts checklist | 2 |
| **INTRODUCTION** | | | | |
| *Background* | 3a | D;E | Explain the healthcare context (including whether diagnostic or prognostic) and rationale for developing or evaluating the prediction model, including references to existing models | 4,5 |
|  | 3b | D;E | Describe the target population and the intended purpose of the prediction model in the context of the care pathway, including its intended users (e.g., healthcare professionals, patients, public) | 4,5 |
|  | 3c | D;E | Describe any known health inequalities between sociodemographic groups | N/A |
| *Objectives* | 4 | D;E | Specify the study objectives, including whether the study describes the development or validation of a prediction model (or both) | 4,5 |
| **METHODS** | | | | |
| *Data* | 5a | D;E | Describe the sources of data separately for the development and evaluation datasets (e.g., randomised trial, cohort, routine care or registry data), the rationale for using these data, and representativeness of the data | 5,6,7 |
|  | 5b | D;E | Specify the dates of the collected participant data, including start and end of participant accrual; and, if applicable, end of follow-up | 5,6,7 |
| *Participants* | 6a | D;E | Specify key elements of the study setting (e.g., primary care, secondary care, general population)  including the number and location of centres | 5 |
|  | 6b | D;E | Describe the eligibility criteria for study participants | 5 |
|  | 6c | D;E | Give details of any treatments received, and how they were handled during model development or evaluation, if relevant | N/A |
| *Data preparation* | 7 | D;E | Describe any data pre-processing and quality checking, including whether this was similar across  relevant sociodemographic groups | 5-8 |
| *Outcome* | 8a | D;E | Clearly define the outcome that is being predicted and the time horizon, including how and when assessed, the rationale for choosing this outcome, and whether the method of outcome assessment is  consistent across sociodemographic groups | 9-10 |
|  | 8b | D;E | If outcome assessment requires subjective interpretation, describe the qualifications and demographic characteristics of the outcome assessors | N/A |
|  | 8c | D;E | Report any actions to blind assessment of the outcome to be predicted | N/A |
| *Predictors* | 9a | D | Describe the choice of initial predictors (e.g., literature, previous models, all available predictors) and  any pre-selection of predictors before model building | 9,10 |
|  | 9b | D;E | Clearly define all predictors, including how and when they were measured (and any actions to blind assessment of predictors for the outcome and other predictors) | 9,10 |
|  | 9c | D;E | If predictor measurement requires subjective interpretation, describe the qualifications and demographic characteristics of the predictor assessors | 9,10 |
| *Sample size* | 10 | D;E | Explain how the study size was arrived at (separately for development and evaluation), and justify that  the study size was sufficient to answer the research question. Include details of any sample size calculation | 7,8 |
| *Missing data* | 11 | D;E | Describe how missing data were handled. Provide reasons for omitting any data | 6 |
| *Analytical methods* | 12a | D | Describe how the data were used (e.g., for development and evaluation of model performance) in the analysis, including whether the data were partitioned, considering any sample size requirements | 7,8 |
|  | 12b | D | Depending on the type of model, describe how predictors were handled in the analyses (functional form,  rescaling, transformation, or any standardisation). | 8,9 |
|  | 12c | D | Specify the type of model, rationale^2^, all model-building steps, including any hyperparameter tuning,  and method for internal validation | 9 |
|  | 12d | D;E | Describe if and how any heterogeneity in estimates of model parameter values and model performance was handled and quantified across clusters (e.g., hospitals, countries). See TRIPOD-Cluster for  additional considerations^3^ | 9, 10 |
|  | 12e | D;E | Specify all measures and plots used (and their rationale) to evaluate model performance (e.g., discrimination, calibration, clinical utility) and, if relevant, to compare multiple models | 9, 10 |
|  | 12f | E | Describe any model updating (e.g., recalibration) arising from the model evaluation, either overall or for particular sociodemographic groups or settings | N/A |
|  | 12g | E | For model evaluation, describe how the model predictions were calculated (e.g., formula, code, object, application programming interface) | 9,10 |
| *Class imbalance* | 13 | D;E | If class imbalance methods were used, state why and how this was done, and any subsequent methods to  recalibrate the model or the model predictions | 15, 16 |
| *Fairness* | 14 | D;E | Describe any approaches that were used to address model fairness and their rationale | 15, 16 |
| *Model output* | 15 | D | Specify the output of the prediction model (e.g., probabilities, classification). Provide details and  rationale for any classification and how the thresholds were identified | 13-16 |

^1^ D=items relevant only to the development of a prediction model; E=items relating solely to the evaluation of a prediction model; D;E=items applicable to both the development and evaluation of a prediction model

^2^ Separately for all model building approaches.

^3^ TRIPOD-Cluster is a checklist of reporting recommendations for studies developing or validating models that explicitly account for clustering or explore heterogeneity in model performance (eg, at different hospitals or centres). Debray et al, BMJ 2023; 380: e071018 [DOI: 10.1136/bmj-2022-071018]

| *Training versus*  *evaluation* | 16 | D;E | Identify any differences between the development and evaluation data in healthcare setting, eligibility  criteria, outcome, and predictors | N/A |
| --- | --- | --- | --- | --- |
| *Ethical approval* | 17 | D;E | Name the institutional research board or ethics committee that approved the study and describe the participant-informed consent or the ethics committee waiver of informed consent | 24 |
| **OPEN SCIENCE** | | | | |
| *Funding* | 18a | D;E | Give the source of funding and the role of the funders for the present study | 24 |
| *Conflicts of interest* | 18b | D;E | Declare any conflicts of interest and financial disclosures for all authors | 24 |
| *Protocol* | 18c | D;E | Indicate where the study protocol can be accessed or state that a protocol was not prepared | 24 |
| *Registration* | 18d | D;E | Provide registration information for the study, including register name and registration number, or state  that the study was not registered | N/A |
| *Data sharing* | 18e | D;E | Provide details of the availability of the study data | 25 |
| *Code sharing* | 18f | D;E | Provide details of the availability of the analytical code^4^ | 25 |
| **PATIENT & PUBLIC INVOLVEMENT** | | | | |
| *Patient & Public Involvement* | 19 | D;E | Provide details of any patient and public involvement during the design, conduct, reporting, interpretation, or dissemination of the study or state no involvement. | N/A |
| **RESULTS** | | | | |
| *Participants* | 20a | D;E | Describe the flow of participants through the study, including the number of participants with and without the outcome and, if applicable, a summary of the follow-up time. A diagram may be helpful. | 11 |
|  | 20b | D;E | Report the characteristics overall and, where applicable, for each data source or setting, including the key dates, key predictors (including demographics), treatments received, sample size, number of outcome events, follow-up time, and amount of missing data. A table may be helpful. Report any  differences across key demographic groups. | 11 |
|  | 20c | E | For model evaluation, show a comparison with the development data of the distribution of important predictors (demographics, predictors, and outcome). | N/A |
| *Model development* | 21 | D;E | Specify the number of participants and outcome events in each analysis (e.g., for model development, hyperparameter tuning, model evaluation) | 7-10 |
| *Model specification* | 22 | D | Provide details of the full prediction model (e.g., formula, code, object, application programming interface) to allow predictions in new individuals and to enable third-party evaluation and implementation, including any restrictions to access or re-use (e.g., freely available, proprietary)^5^ | 8,9, 25 |
| *Model performance* | 23a | D;E | Report model performance estimates with confidence intervals, including for any key subgroups (e.g., sociodemographic). Consider plots to aid presentation. | 10 |
|  | 23b | D;E | If examined, report results of any heterogeneity in model performance across clusters. See TRIPOD  Cluster for additional details^3^. | N/A |
| *Model updating* | 24 | E | Report the results from any model updating, including the updated model and subsequent performance | N/A |
| **DISCUSSION** | | | | |
| *Interpretation* | 25 | D;E | Give an overall interpretation of the main results, including issues of fairness in the context of the  objectives and previous studies | 17-22 |
| *Limitations* | 26 | D;E | Discuss any limitations of the study (such as a non-representative sample, sample size, overfitting, missing data) and their effects on any biases, statistical uncertainty, and generalizability | 19, 20 |
| *Usability of the model in the context of current care* | 27a | D | Describe how poor quality or unavailable input data (e.g., predictor values) should be assessed and handled when implementing the prediction model | 21, 22 |
|  | 27b | D | Specify whether users will be required to interact in the handling of the input data or use of the model,  and what level of expertise is required of users | N/A |
|  | 27c | D;E | Discuss any next steps for future research, with a specific view to applicability and generalizability of  the model | 22, 23 |

1. **Python Libraries and packages utilized in this Project:**
2. scikit-Learn v.1.2.2: scikit-learn.org/stable/install.html
3. combat v0.3.3: epigenelabs.github.io/pyComBat/
4. gffutils v0.12: github.com/daler/gffutils
5. neuroCombat v0.2.12: github.com/Jfortin1/neuroCombat
6. matplotlib v3.9: matplotlib.org/stable/
7. Hotelling’s T2 ellipse from scipy v1.9: scipy.org; matplotlib v3.9: matplotlib.org/stable/

**
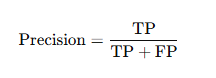
Formulas:**

**Precision:**

**
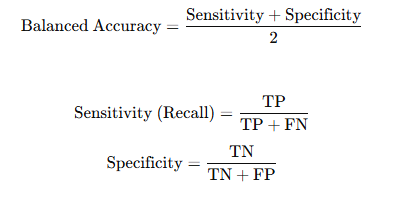
**

**Balanced Accuracy:**

**
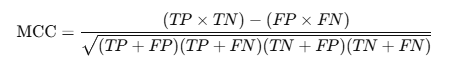
**

**MCC:**

TP: true positive, TN: true negative, FP: false positive, FN: false negative

_
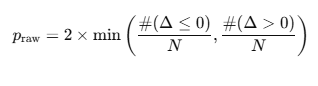
_**Raw p-value from bootstrapping:**

Metric difference**:** Δ=metric_modelA_ − metric_modelB_

_where: N = number of bootstrap interations; (two-tailed: testing difference (high or low))_

**
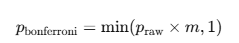
Bonferroni Correction:**

m = total number of pairwise comparisons for that metric (for shared labels (4 models): m = 6 pairwise comparisons; for unique labels (2 models): m= 1 ))

**Supplemental References:**

1. Carré A, Klausner G, Edjlali M, et al. Standardization of brain MR images across machines and protocols: bridging the gap for MRI-based radiomics. Sci Rep. 2020;10(1):12340. Published 2020 Jul 23. doi:10.1038/s41598-020-69298-z
2. Stamoulou E, Spanakis C, Manikis GC, Karanasiou G, Grigoriadis G, Foukakis T, Tsiknakis M, Fotiadis DI, Marias K. Harmonization Strategies in Multicenter MRI-Based Radiomics. J Imaging. 2022 Nov 7;8(11):303. doi: 10.3390/jimaging8110303. PMID: 36354876; PMCID: PMC9695920.
3. Wang Z et al. Image quality assessment: from error visibility to structural similarity. IEEE Transactions on Image Processing. 2004;13(4):600-612.
4. DeGroot, Morris H. Probability and Statistics (2nd ed.) 1980. Addison-Wesley.
5. Faragallah OS et al. A comprehensive survey analysis for present solutions of medical image fusion and future directions. IEEE Access. 2021;9:11358-11371. doi: 10.1109/ACCESS.2020.3048315
6. Alqutayfi A, Al-azani S. A comparative study of generative models for T1-to-T2 reconstruction. arXiv. 2026:2602.07068. https://doi.org/10.48550/arXiv.2602.07068
7. Tejani AS, Klontzas ME, Gatti AA, et al. Checklist for Artificial Intelligence in Medical Imaging (CLAIM): 2024 Update. Radiol Artif Intell. 2024;6(4):e240300. doi:10.1148/ryai.240300
8. Collins GS, Moons KGM, Dhiman P, et al. *BMJ* 2024;385:e078378. doi:10.1136/bmj-2023-078378
